# Effect of temperature and time after collection on buck sperm quality

**DOI:** 10.1186/s12917-019-2135-y

**Published:** 2019-10-22

**Authors:** Kirsten Hahn, Klaus Failing, Axel Wehrend

**Affiliations:** 1 Equine Hospital, Ludwig-Maximilians-University, Veterinärstraße 13, 80539 Munich, Germany; 2 Unit for Biomathematics and Data Processing, Justus-Liebig University, Giessen, Germany; 3 Clinic for Obstetrics, Gynecology and Andrology of Large and Small Animals with Ambulatory Service, Justus-Liebig-University, Giessen, Germany

**Keywords:** Peacock bucks, Temperature, Time point, Computer assisted semen analysis

## Abstract

**Background:**

Different parameters are assessed as part of the semen analysis but a standard protocol for evaluation of goat semen is still missing. The aim of this study was to analyse two different factors affecting buck sperm quality in the post-collection period prior to adding the extender. Here we examined the effects of two handling temperatures (20 °C, 37 °C) and various examination time points (3–30 min) after semen collection.

**Results:**

Examination time point had a significant influence on raw sperm viability (*p* < 0.05), motility (*p* < 0.05) and on semen pH (*p* < 0.05). The two different handling temperatures had no significant effect on sperm viability (*p* > 0.05), motility (*p* > 0.05), with the exception of fast moving sperm (*p* = 0.04), or on semen pH (*p* > 0.05).

**Conclusion:**

Examination time point was identified as factor strongly influencing raw peacock buck semen after collection. Raw goat semen can tolerate room temperatures for at least 10 min without impacting overall semen quality. In order to obtain comparable results, semen samples should always be examined within 10 min after collection.

## Background

Different parameters are assessed as part of the semen analysis including in general volume, color, consistency, impurities as well as semen concentration, motility, vitality and morphology [6]. These are used to determine the suitability of an ejaculate for further use and to establish an insemination dose. Therefore, the handling conditions must be kept as constant as possible during the evaluation. In human medicine, guidelines for semen collection, handling and evaluation of the ejaculates are described [33].

A standard protocol for evaluation of goat semen does not exist. Usually the semen evaluation takes place directly after the collection [26]. In other studies the time interval between collection and evaluation was 2–3 min [29], after 20 min [28] or for the entire evaluation within one hour after collection, while concentration, pH and volume were examined immediately after collection [5]. Busch and Fischer (2007) suggested that the collected semen must be evaluated within 10 min after collection, without describing the influence of time on semen quality [6].

The handling temperature during semen evaluation is usually 37 °C [5, 7, 16, 25, 26, 29]. All materials having contact with the sperm should be preheated to this temperature [9, 27].

The sperm of mammals are very sensitive to temperature fluctuations [18, 20] with species and individual differences. Equine semen should be kept at 37 °C prior to dilution [14]. It is known that boar spermatozoa are very susceptible to cold shock, especially when stored lower than 15 °C [12]. Sperm motility was better for undiluted semen samples stored at 15 °C and 20 °C for 48 h compared to 4 °C and 39 °C [34]. In literature different handling temperatures for goat spermatozoa after collection are described varying from 30 °C to 37 °C [6, 19, 28]. In general, semen is usually preserved at 18–22 °C or at 37 °C until adding an extender [4].

In ruminants pH of semen is in the slightly acidic with range of 6.4–7.0. Deviations may be due to accessory gland disease and can have an adverse effect on semen viability [31].

At present, however, only a limited number of studies have assessed the factors that can influence the semen analysis results of raw goat semen. Although different studies have evaluated the influence of temperature on semen quality in mammal species [3, 17] systematic investigations on caprine semen in this critical post-collection period prior to adding an extender are still missing.

The aim of this study was to investigate two possible factors affecting buck sperm quality after collection prior to adding an extender. Therefore, we evaluated the influence of two different post-collection temperatures (20 °C and 37 °C) and various examination time points (3–30 min) after semen collection on semen quality.

## Results

### Macroscopic evaluation

The volume of the ejaculates was on average 0.48 ml ± 0.11 ml (minimum: 0.3 ml, maximum: 0.8 ml). The majority (80%) of all collected semen samples (*n* = 20) had a yellow color and 20% were ivory. Consistency was milky in 70% of the ejaculates and creamy in 30%.

### pH value

After semen collection, pH in 65% (*n* = 13) of the samples were within the reference range (6.4–7.0) for the pH value of goat semen [31]. About 35% (*n* = 7) had a slightly increased pH value of 7.2. All results are shown in Table 1 for both handling temperatures overtime.

**Table 1:** pH values of fresh ejaculates (*n* = 20) of five peacock bucks at storage temperatures of 20 °and 37 °C at the specified examination times. Presented by arithmetic mean (x̄), standard deviation (SD), minimum and maximum (range)

Time (min.)	Temperature (°C)	x̄ ± SD	Range
0	20 and 37	7.02 ± 0.19	6.4–7.2
10	20	6.97 ± 0.20	6.4–7.2
	37	6.83 ± 0.29	6.4–7.7
20	20	6.90 ± 0.20	6.4–7.2
	37	6.70 ± 0.33	< 6.4–7.7
30	20	6.81 ± 0.21	6.4–7.2
	37	6.55 ± 0.36	< 6.4–7.5
40	20	6.79 ± 0.19	6.4–7.0
	37	6.50 ± 0.37	< 6.4–7.5
50	20	6.74 ± 0.26	< 6.4–7.0
	37	6.42 ± 0.29	< 6.4–7.0
60	20	6.69 ± 0.24	< 6.4–7.0
	37	6.42 ± 0.29	< 6.4–7.0

Semen pH was significantly affected by both examination time point (*p* < 0.0001) and temperature (*p* = 0.002). Further the interaction between duration of storage and handling temperature (*p* < 0.0001) indicated that the pH decreased faster over time when stored at 37 °C.

### Microscopic evaluation

Semen viability and morphological abnormalities.

**Fig. 1:**
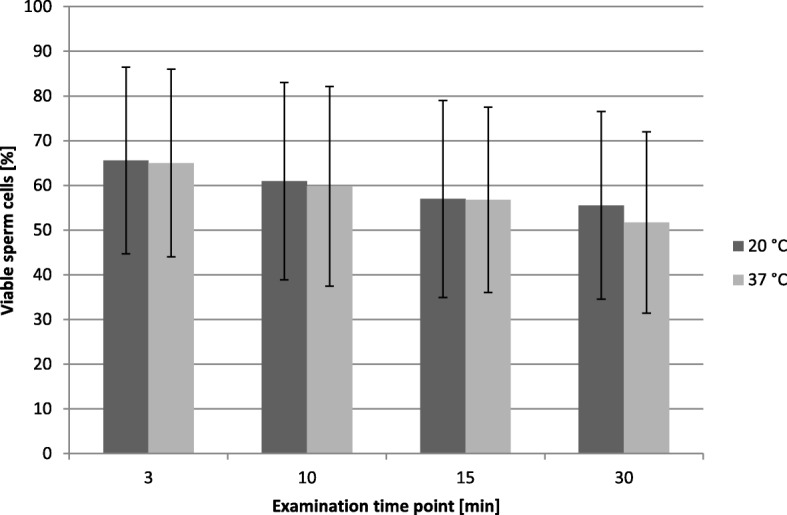
Proportion of viable sperm cells (%) in native ejaculates (*n* = 20) of five peacock bucks compared at post-collection temperatures of 20 ° and 37 °C at different examination times (x̄ ± SD)

The results of semen viability for both handling temperatures and the different examination time points are shown in Fig. 1.

Examination time points influenced proportion of living and dead sperm cells (*p* < 0.0001) whereas the effect of the handling temperature as well as its interaction to the examination time point did not have a significant effect (*p* = 0.308 resp. *p* = 0.458). The results are also presented in Table 2. Living and dead sperm were distinguished by their color. Sperm without stain were alive whereas the blue sperm were determined as dead.

**Table 2:** Results of the two-way ANOVA with repeated measures for the analyzed influencing factors are presented by their *p*-values

Parameter	Factor		Interaction
	Temperature	Time	Temperature x Time
Viability	0.30	< 0.0001	0.45
pH	0.002	< 0.0001	< 0.0001
Motility	0.13	0.001	0.06
Progressive motility	0.13	0.001	0.06
Fast motility	0.04	< 0.0001	0.05
Slow motility	0.83	0.69	0.09
Circle motility	0.45	0.07	0.0003
Immotile	0.13	0.001	0.06

The proportion of morphological abnormalities was on average 2.80 ± 3.45% at a storage temperature of 20 °C, and 2.45 ± 2.83% at 37 °C. The results for all sperm with morphological abnormalities for both storage temperatures are presented in Table 3. This was a single evaluation taken at 3 min.

**Table 3:** Morphological abnormalities of sperm cells from all peacock bucks (*n* = 5) of all collected ejaculates (*n* = 20) presented by arithmetic mean (x̄), standard deviation (SD), minimum and maximum (range). This was a single evaluation made at 3 min after collection

Parameters	Temperature (°C)	x̄ ± SD	Range
Head defects	20	0.17 ± 0.33	0–1
	37	0.25 ± 0.30	0–1
Tail defects	20	1.45 ± 2.50	0–11
	37	1.20 ± 2.3	0–10.5
Loose Heads	20	1.07 ± 1.61	0–5
	37	0.97 ± 1.15	0–4.5
Cytoplasmic droplets	20	0.10 ± 0.26	0–1
	37	0.25 ± 0.11	0–0.5
Total	20	2.80 ± 3.45	0–15
	37	2.45 ± 2.83	0–13

### Sperm motility

Results of the computer-assisted semen analysis are shown in Table 4 for both temperatures and all examination time points.

**Table 4:** Measurement results of the motility parameters determined by CASA (AndroVision® system) in the ejaculates (*n* = 20) of five peacock bucks at a storage temperature of 20° and 37 °C. Due to the fact that the statistical distribution of the circle motility was skewed to the right this data is presented by arithmetic mean (x̄) and minimum and maximum (range)

Parameter	Temperature (°C)	3 min. (x̄ ± SD)	10 min. (x̄ ± SD)	15 min. (x̄ ± SD)	30 min. (x̄ ± SD)
Progressive motility (%)	20	71.72 ± 24.05	73.16 ± 23.11	72.17 ± 23.61	68.72 ± 24.69
	37	73.84 ± 22.85	67.78 ± 24.14	71.58 ± 27.64	59.90 ± 27.01
Fast motility (%)	20	43.31 ± 26.42	41.43 ± 25.48	40.61 ± 25.99	37.37 ± 25.04
	37	42.61 ± 27.82	36.99 ± 25.81	39.97 ± 26.16	28.18 ± 24.06
Slow motility (%)	20	27.71 ± 11.58	30.66 ± 17.02	30.55 ± 15.23	30.37 ± 14.40
	37	29.60 ± 16.58	29.91 ± 11.32	30.82 ± 17.59	31.10 ± 14.30
Circle motility (%)	20	0.70 (range: 0–2.9)	1.07 (range: 0–4.4)	1.02 (range: 0–4.6)	0.98 (range: 0–3.9)
	37	1.23 (range: 0–10.9)	0.87 (range: 0–3.8)	0.78 (range: 0–5.5)	0.62 (range:0–4.3)
Immotile (%)	20	28.28 ± 24.05	26.84 ± 23.11	27.83 ± 23.61	31.28 ± 24.69
	37	26.15 ± 22.85	32.22 ± 24.14	28.42 ± 27.65	40.09 ± 27.01

The two different handling temperatures had no significant effect on sperm motility (*p* > 0.05) with exception of fast motile sperm (*p* = 0.04). Sperm with a VCL < 90 were defined as slow motile and with a VCL ≥ 90 are defined as fast motile according to the CASA settings. Examination time point was identified as factor influencing semen motility (*p* = 0.0012) and especially fast motile (progressive) spermatozoa (*p* < 0.0001). Interaction of sample handling temperature and time had a significant influence on the proportion of spermatozoa that are moving in circles (*p* = 0.0003). All data of the two-way ANOVA are presented in Table 2. Essentially, the results of glmm correspond to the two-way ANOVA.

## Discussion

Various factors are known to influencing goat semen quality such as age, breed, season, method of semen collection, extender and centrifugation [1, 2, 11, 15, 29]. In our study we investigated post-collection temperature and time as two possible factors influencing buck semen quality after collection with a focus on raw semen.

Sperm motility is an important parameter which influences fertility of a male animal [13]. Temperature essentially influences semen motility. In one study with dogs, the examined motility parameters at a temperature of 30 degrees were significantly lower compared to 37 degrees [30]. Tuli and Holz (1995) recommend examining sperm motility in isothermal conditions of 36–38 °C. Verstegen et al. (2002) suggested 37 °C is an ideal temperature for semen evaluation.

These recommendations do not seem to be applied to raw semen of peacock goat bucks, according to the results of our study. After assessing the measurements of the CASA system, there was a tendency that individual motility of the semen cells was higher at a post-collection temperature of 20 °C imitating room temperature. A significant influence of temperature was observed on the proportion of fast-motile sperm again with better results when kept at 20 °C. According to our results goat semen can be kept at room temperature after collection and during semen analysis without affecting overall semen quality. However, we compared only two different temperatures in the work. Further studies would be necessary to investigate a larger number of different handling temperatures for raw goat semen.

The interaction effect between post-collection temperature and time of investigation had a significant effect on sperm that exhibited circling in both statistical evaluation methods. With the computer assisted semen analysis, further various movement parameters of sperm can be assessed. To date, studies on the influence on the proportion of spermatozoa moving in circles are missing. According to Pezzanite et al. [23], sperm moving in circles belong to the category of immotile sperm. Waberski and Petrunkina [31] include spermatozoa with circle motility, as long as the circle corresponds at most to the sperm head lengths, to the progressive motile sperm. Possible causes of the circular movement are pathological tail changes, premature hyper-activation, as well as osmotic changes, which can lead to a curling of the flagella.

In addition, the influence of handling temperature on semen viability was investigated. No influence could be demonstrated. According to our results fresh peacock goat semen can be kept at 20 °C or 37 °C after collection without influence on semen viability. Murphy et al. [21] also demonstrated no effect of different storage temperatures (5, 15, 22, 32 °C) on liquid bull semen viability.

According to Busch and Fischer (2007) undiluted semen of small ruminants kept at 30 °C post collection for 20–30 min can still be used for artificial insemination. This statement is comparable to the results in our work. At a post-collection temperature of 20 °C, the obtained ejaculates still achieved the minimum requirements [7] placed on fresh goat semen. The ejaculates kept at 37 °C were just below the minimum requirements.

For semen quality, a stable pH is essential. If values below 6.5 are reached, motility and metabolism of the sperm are gradually reduced [32]. In this work, pH of the semen was stable when the samples were kept at a temperature of 20 °C. At both post-collection temperatures used, however, a steady decrease of the pH was observed with increasing duration of the tests. Even after 10 min, a lowering of the pH value could be determined in both experimental batches, but pH decreased faster at 37 °C. These pH shifts result from aerobic and anaerobic metabolic products of living and dead sperm. Perhaps there is a relationship between decreasing pH and decreasing sperm viability. There are individual differences between the bucks with regard to the pH of the semen. Possibly the individual buck also influences the stability of the pH value in the raw ejaculate.

Additionally we investigated different examination time points as second influencing factor on goat semen quality. Different data exists regarding the time of semen examination after collection. As a rule, the semen examination is carried out directly after collection [22, 26]. In other studies, the examination time differed between 2 and 3 min [29], after 20 min [28] or within 1 h after collection [5, 7, 10].

Examination time significantly influenced semen motility. Especially fast motility was affected and slightly decreased between the examination time points 3 and 10 min. Our results accord to Busch and Fischer (2007) who recommended that the extracted ejaculate must be examined within 10 min after collection.

The proportion of live sperm decreases with an increasing duration of storage time. Therefore, this parameter should generally be examined as soon as possible after semen collection. In both statistical evaluation methods used here, these observations could be confirmed by the achievement of the given level of significance.

The time had a significant influence on the pH value of the fresh semen samples. At the later examination times, a decrease in the pH value could be observed for all goat bucks. The time of examination is therefore important for semen pH and therefore for the quality of spermatozoa, since the motility and the metabolism can be reduced. The examination and further processing of the sperm should be carried out as soon as possible after collection, since otherwise semen quality can be adversely affected by a reduced pH value.

Other conditions (aerobic versus anaerobics, light versus dark, presence of urine) play an important role for semen quality. In order to obtain comparable results, it is necessary to use a set of standard conditions. Further studies are needed for an optimal standard protocol for evaluation of goat semen.

## Conclusion

Examination time point was identified as factor influencing fresh peacock goat semen assessment. Semen motility, viability and pH should be assessed within 10 min after semen collection. Raw goat semen can tolerate room temperatures for at least 10 min without impacting overall semen quality. In order to obtain comparable results, the same examination time should always be chosen. Andromed®, a commercial semen extender without animal proteins, was useful for goat semen analysis with the CASA system. Handling temperature influenced fast motile sperm as well as semen pH, with a tendency of slightly better results for both parameters at 20 °C.

## Methods

### Animals

Five clinically and reproductive healthy peacock goats were used for this study (age < 12 months). All bucks were owned, maintained and managed at the Clinic for Obstetrics, Gynecology and Andrology of Large and Small Animals with Ambulatory Service of the Justus-Liebig-University Giessen. The goats were housed in groups under natural light and were given hay, mineral supplement and fresh water ad libitum. The study was performed during January – April 2015. All experimental procedures were approved by the Ethics Committee of Regierungspraesidium Gießen Germany (Approval number A 27/2012). After the study, the animals were still in the possession of the clinic.

### Semen collection

Prior to the beginning of this study, all bucks were trained to use of a special artificial vagina prepared for sheep and goats (Fa. Minitube, Tiefenbach) containing water at 41 °C. We used two different female Alpine goats independent of their stage of cycle for sexual stimulation of the bucks. All semen collections were performed under the same conditions in a separate room. A total of 20 ejaculates (mean 4 ± 2 per buck) were obtained. Semen was collected (one ejaculate per buck per collection day) once per week with a break of at least 5 days.

### Semen evaluation

Macroscopic evaluation (volume, color, consistency, smell, impurities) of all ejaculates was performed directly after semen collection. The pH of each semen sample was measured with pH indicator-paper calibrated with whole numbers (Fa. Merck group, Darmstadt) every 10 min for 1 h following collection. Concentration and sperm motion parameters were evaluated using computer assisted semen analysis (CASA, AndroVision®-System, Fa. Minitube, Tiefenbach) on a plate warmed to 38 °C, negative phase contrast and × 10 objective at 3, 10, 15 and 30 min after collection. Due to the high sperm concentration, all samples had to be diluted just before evaluation at each time point for computer analysis (1:29). The extender was previously kept at 20 °C or 37 °C. The amount of extender depended on the sperm concentration. For this purpose a commercial semen extender without animal proteins was used (Andromed® extender, Fa. Minitube, Tiefenbach). Diluted semen samples were filled into Leja® counting chamber slides (Fa. LabIVF, Singapore) and directly a minimum of 2000 sperm cells were analysed. The technical setting parameters of the CASA system are presented in Table 5. Evaluation of semen viability and morphological abnormalities was performed using the bromophenol-nigrosin staining method [8, 24]. After air-drying, the smear was observed under a phase-contrast microscope (1000x) and for each, 200 sperm were evaluated.

**Table 5:** Technical settings of the CASA system AndroVision® version 1.0.0.5; 2012 for motility analysis of buck spermatozoa

Variables	Settings
Depth of sample chamber	20 μm
Temperature during analysis	38 °C
Total number of cells evaluated	2000 spermatozoa
Sperm recognition area	10–100 μm^2^
Frame rate	60 frames/s
Pixel/μm	1/0.54
Progressive motility	Every cell that is not “immotile” or “local motile”
Immotile	VSL < 12.0 and ALH < 1.50
Local motility	VCL < 60.0 and VSL < 48.0
Circle motility	Radius > 9.0 and radius < 90.0 and rotation > 0.70
Slow motility	VCL < 90.0
Fast motility	others

*Abbreviations*: *VCL* Velocity curved line (μm/s), *VSL* Velocity straight line (μm/s), *ALH* Amplitude of lateral head displacement (μm)

### Experimental design

Influence of handling temperature after collection on semen quality.

After semen collection two semen aliquots (each 100 μl) were taken from the original sample and aliquots were kept at two different handling temperatures (20° and 37 °C) for further evaluation.

### Influence of evaluation time on semen quality

Microscopic examination was carried out 3, 10, 15 and 30 min after semen collection, except for morphological abnormalities, which were only examined once at 3 min post semen collection. The pH of each semen sample was measured every 10 min for 1 h following collection.

### Statistical analysis

All data were analysed using the statistical program packages BMDP/Dynamic, Release 8.1 (1993; BMDP Statistical Software, Inc.) and program package R 3.1.2 (2014; Free Software Foundations GNU project, R-package Ime4 R-Function Imer) In accordance to the design of the experiment, two-way ANOVA with repeated measures was applied to test the effects of handling temperature and of the time after semen collection as well as their interaction. In the data pseudo-replications were present (some different ejaculates from the identical buck) but the ANOVA could not take the hierarchical structure of the samples into account, in addition. Despite the low sample size per buck, additionally an asymptotic generalized linear mixed model analysis (glmm) with the statistical program package R 3.1.2 was performed to validate the ANOVA results. In case of the morphological sperm abnormalities the statistical distribution of the data was strongly skewed to the right and represents count information. Therefor this data were analyzed with a Poisson regression model using the statistical program package R.

In general, results were considered statistically significant at *p* ≤ 0.05.

## Data Availability

The datasets used and/or analysed during the current study are available from the corresponding author on reasonable request.

## Abbreviations

ANOVA: Analysis of variance
CASA: Computer assisted semen anaylsis
Max: Maximum
Min: Minimum
pH: Decimal logarithm of the reciprocal of the hydrogen ion activity
